# Extracellular Tumor-Related mRNA in Plasma of Lymphoma Patients and Survival Implications

**DOI:** 10.1371/journal.pone.0008173

**Published:** 2009-12-15

**Authors:** Vanesa Garcia, Jose Miguel Garcia, Javier Silva, Paloma Martin, Cristina Peña, Gemma Dominguez, Raquel Diaz, Mercedes Herrera, Constanza Maximiano, Pilar Sabin, Antonio Rueda, Miguel Angel Cruz, Jose Rodriguez, Miguel Angel Canales, Felix Bonilla, Mariano Provencio

**Affiliations:** 1 Department of Medical Oncology, Puerta de Hierro University Hospital, Madrid, Spain; 2 Department of Molecular Pathology, Puerta de Hierro University Hospital, Madrid, Spain; 3 Department of Medical Oncology, Gregorio Marañón Hospital, Madrid, Spain; 4 Department of Medical Oncology, Virgen de la Victoria Hospital, Málaga, Spain; 5 Department of Medical Oncology, Virgen de la Salud Hospital, Toledo, Spain; 6 Department of Hematology, La Paz Hospital, Madrid, Spain; Duke-NUS Graduate Medical School, Singapore

## Abstract

**Background:**

We studied anomalous extracellular mRNAs in plasma from patients with diffuse large B-cell lymphoma (DLBCL) and their survival implications. mRNAs studied have been reported in the literature as markers of poor (*BCL2*, *CCND2*, *MYC*) and favorable outcome (*LMO2*, *BCL6*, *FN1*) in tumors. These markers were also analyzed in lymphoma tissues to test possible associations with their presence in plasma.

**Methodology/Principal Findings:**

mRNA from 42 plasma samples and 12 tumors from patients with DLBCL was analyzed by real-time PCR. Samples post-treatment were studied. The immunohistochemistry of BCL2 and BCL6 was defined. Presence of circulating tumor cells was determined by analyzing the clonality of the immunoglobulin heavy-chain genes by PCR. In DLBCL, *MYC* mRNA was associated with short overall survival. mRNA targets with unfavorable outcome in tumors were associated with characteristics indicative of poor prognosis, with partial treatment response and with short progression-free survival in patients with complete response. In patients with low IPI score, unfavorable mRNA targets were related to shorter overall survival, partial response, high LDH levels and death. mRNA disappeared in post-treatment samples of patients with complete response, and persisted in those with partial response or death. No associations were found between circulating tumor cells and plasma mRNA. Absence of BCL6 protein in tumors was associated with presence of unfavorable plasma mRNA.

**Conclusions/Significance:**

Through a non-invasive procedure, tumor-derived mRNAs can be obtained in plasma. mRNA detected in plasma did not proceed from circulating tumor cells. In our study, unfavorable targets in plasma were associated with poor prognosis in B-cell lymphomas, mainly *MYC* mRNA. Moreover, the unfavorable targets in plasma could help us to classify patients with poor outcome within the good prognosis group according to IPI.

## Introduction

DLBCL accounts for approximately 30% of new diagnoses and more than 80% of aggressive lymphomas. Recently, anti-CD20 monoclonal antibody (rituximab) has been combined with CHOP, which has improved survival [1]. DLBCL is a non-uniform subgroup of tumors with a variety of clinical presentations, outcomes, response to treatment and genetic alterations. This could be because DLBCL consists of several diseases, but stratification into subclasses by standard pathologic techniques has not been possible. Thus, studies about individual prognostic markers and prognostic models based on the combination of several markers have been performed, so increasing the insights of molecular heterogeneity [2]. There is disparity between the genes that comprise the predictive models of the studies, which could be due to different criteria for patient selection and statistical algorithms used for their construction [2]. In an attempt to devise a technically simple model, Lossos and colleagues evaluated 36 genes found to be predictive of outcome in gene array studies and created a prediction model validated in these previous studies [3].

Cell-free nucleic acids derived from tumors are detectable in plasma of cancer patients and may show diagnostic and prognostic values [4], [5]. The use of these markers in plasma as a source of tumor information would be important, since they are obtained by a non-invasive method that would be useful during patients' follow-up and treatment monitoring. To our knowledge, there are few studies of lymphoma that analyze nucleic acids in plasma. Epstein-Barr virus DNA, rearranged immunoglobulin heavy chain DNA and telomerase mRNA, in a preliminary study, were detected in plasma from patients with lymphomas [6]–[8]. Thus, the prognostic value of mRNA detected in plasma from DLBCL patients is a novel approach.

This is the first study analyzing mRNA in plasma from patients with DLBCL. In this study, we analyzed plasma mRNA of three “unfavorable” genes, which have been described as markers of poor outcome and components of the activated B-cell signature (*CCND2*, *BCL2* and *MYC*), and three “favorable” genes, which have been described as markers of good outcome and components of the germinal-center B-cell signature (*BCL6* and *LMO2*) and lymph-node signature (*Fibronectin 1*). These unfavorable and favorable markers (both mRNA and protein) have been studied in individually and/or in a large data set in DLBCL tissues and have been associated with shorter and longer survival, respectively [2]. In the study of Lossos and colleagues [3] evaluating 36 genes, our markers showed high absolute univariate z scores. Analysis of plasma mRNA of the six genes was performed using real-time polymerase chain reaction (RT-PCR). We investigated whether these markers in plasma were associated with progression-free survival (PFS), overall survival (OS), a series of clinico-pathological parameters and treatment response. The possible relation between plasma mRNA and circulating tumor B-cells was studied. Moreover, we analyzed the levels of mRNA and BCL2 and BCL6 protein in DLBCL tumor tissues by PCR and immunohistochemistry (IHC), respectively.

## Materials and Methods

### Patients and Clinical Characteristics

We recruited prospectively 42 DLBCL patients with a median follow-up of 13.7 months (range: 3–35 months), 25 Follicular Lymphoma (FL) cases and 16 with Hodgkin's lymphoma (HL). Samples were provided mainly by Puerta de Hierro Majadahonda University Hospital, with the collaboration of four other Spanish hospitals. Informed written consent was obtained from all participants after an explanation of the nature of the study, as approved by the research ethics board of Puerta de Hierro Majadahonda University Hospital acting as reference ethics board for the other five hospitals, in line with Spanish legislation in cases of collaborative research studies. The histological diagnosis was ascertained by lymph node biopsy. Blood samples were taken before the treatment began. Between 4 and 6 weeks after treatment, blood samples were taken from 13 patients with DLBCL. Blood samples from 50 healthy blood donors were obtained at the hematology unit of our hospital. Paraffin blocks from 26 DLBCL tumors were selected only on the basis of the availability of suitable formalin-fixed, paraffin-embedded tissue. Twelve out of 26 blocks had enough material to extract RNA.

At diagnosis, the following data were available: age, sex, stage, histological grade, “B” symptoms, number of affected lymph node regions, extranodal involvement, bone marrow infiltration, performance status, bulky disease, detection of Hepatitis C Virus, standard hematological tests and levels of LDH, β_2_-microglobulin and albumin. The International Prognostic Index (IPI) and FLIPI (for FL) scores were determined.

### Clinical Follow-Up and Treatment

Prospective follow-up, starting after diagnosis and treatment, was based on regular (every 3 months during the first and second year, every 6 months during the third year, and then yearly until relapse) clinical, biochemical and radiological examinations (chest and abdominal CT), gallium scan and MNR or PET if suggested by radiologists. OS and PFS were defined as the period from time of diagnosis until death and the interval between the start of treatment and disease progression or recurrence. In DLBCL, 32 patients received CHOP-Rituximab (CHOP-R) and 10 patients received CHOP-R plus radiotherapy. In FL, 14 patients were treated with CHOP-R, 3 with CVP-R, 2 with radiotherapy, 2 with CHOP-R plus radiotherapy and 4 patients with low tumor burden were managed with a “watch and wait” policy. In HDK, 8 patients received chemotherapy (ABVD), one received radiotherapy and 7 received ABVD plus radiotherapy. CHOP-R consists of cyclophosphamide, doxorubicin, vincristine, prednisone and rituximab, administered to the standard doses for this regimen. CVP-Rituximab is like the previous regimen without doxorubicin. The ABVD regimen consists of adriamycin, bleomycin, vinblastine and dacarbazine.

### Plasma Processing, mRNA Isolation and Real-Time PCR

Blood samples were stored at 4°C for 24 hours and were subsequently processed by two centrifugations (300×g and 2,000×g for 30 min each one). Plasma samples were carefully divided in aliquots of 1 ml and snap frozen at −80°C until processing.

mRNA was obtained from 1 ml of plasma by Dynabeads mRNA DIRECT Kit. Plasma was incubated with 100 µl of Dynabeads Oligo (dT) for 10 min at room temperature, and mRNA eluted in 10 mM Tris-HCl. mRNA from 12 paraffin-embedded tumors, was isolated by RNeasy FFPE Kit (Qiagen Inc., Hilden, Germany), according to the manufacturer's instructions. mRNA was reverse-transcribed with the Gold RNA PCR Core Kit (PE Biosystems, Foster City, CA) using random hexamers, according to the manufacturer's instructions.

For RT-PCR, *PGK1, SDHA* and *UBC* (common housekeeping in tumor studies) were evaluated, but their expression in plasma was not stable. Recently, an active release mechanism has been suggested to explain the presence of mRNA in plasma [9]–[11], which could explain the inadequacy of *PGK1, SDHA* and *UBC* as housekeeping genes in studies that determine free mRNA in plasma of cancer patients. In the absence of housekeeping genes, quantitative data cannot be analyzed. For this reason, we analyzed the target mRNAs as presence or absence in plasma. For the study of tumor tissues, mRNA levels were normalized by using *Phosphoglycerate kinase 1* (*PGK1*) as housekeeping gene. RT-PCR was performed in a LightCycler 480 Instrument using the LightCycler 480 SYBR Green I Master Kit (Roche Diagnostics, Mannheim, Germany), according to the manufacturer's instructions. Primers are shown in Table S1. mRNA analysis was performed in duplicate. Melting curve analyses and sequencing in an ABI Prism™ 377 DNA sequencer apparatus (PE Applied Biosystems, Foster City, CA) confirmed the generation of the specific PCR product expected.

### Circulating Tumor Cell Analysis

Total mononuclear cells (MNC) were isolated from the blood of study subjects by Ficoll (Lymphoprep, Axis-Shield PoC AS, Norway) density gradient centrifugation. DNA was obtained by DNeasy Blood and Tissue Kit (Qiagen Inc., Hilden, Germany), according to the manufacturer's instructions. DNA samples obtained from MNC were used to determine clonality of the immunoglobulin heavy-chain genes (IgH) using BIOMED-2 primers by PCR [12]. IgH gene rearrangement was studied, including seven reactions targeting IgH-FR2 (framework region 2) and another seven reactions targeting IgH-FR3 (framework region 3). PCR analysis was done in ABI Genescan (ABI 310 PRISM sequencer), with IgH-FR2 amplification taken as in those cases with a single peak of 250–295 pb and IgH-FR3 amplification, in those cases with a single peak of 100–170 pb.

### Immunohistochemical Analysis

Tumor sections were immunostained with monoclonal antibodies against BCL2 (clone 124; DAKO, Glostrup, Denmark) and BCL6 (clone PG-B6p DAKO, Glostrup, Denmark). Nuclear staining of 30% of neoplastic cells was used to define a case positive for BCL6, whereas the cutoff used to determine BCL2 over-expression was 50% [13].

### Data Analysis

χ^2^ test, Mann-Whitney, Kaplan-Meier and Mantel's log-rank test were used for statistical analysis. *p* values ≤0.05 were considered statistically significant. Statistical analysis used the SPSS, version 13.0 statistical software (SPSS Inc. Chicago, IL).

## Results

### Detection of mRNA in Plasma, Circulating Tumor Cells and Tumor Tissues

In plasma from 42 patients with DLBCL, extracellular mRNA of *CCND2*, *BCL2*, *MYC*, *LMO2*, *BCL6* and *FN1* was detected, respectively, in 14%, 10%, 10%, 10%, 5% and 2% of them. However, no plasma from 50 healthy controls showed presence of all mRNAs, except one case of *CCND2*. Thus, a significant difference was found between patients and controls in *BCL2*, *CCND2*, *MYC* and *LMO2* mRNA (*p* = 0.026, *p* = 0.027, *p* = 0.026 and *p* = 0.026, respectively; χ^2^test; Table S2). The molecular markers were also analyzed in plasma from 25 patients with FL and 16 with HL to test differences with DLBCL, detecting a percentage of positive cases lower than in DLBCL, though the differences are not significant (Table S3). In FL and HL, these markers were not associated with response to treatment and survival.

PCR was performed to determine presence of circulating tumor B-cell monoclonality (FR2, FR3) in MNC from 27 DLBCL patients. Clonality was detected in only one patient from this series (Figure S1), 25 cases were polyclonal and in one case FR3 and FR2 fragments were not amplified. The patient with circulating tumor B-cell monoclonality showed bone marrow infiltration and presence of mRNA in plasma. In the series analyzed, bone marrow infiltration was also detected in another patient, but clonality in MNC was not found. In conclusion, presence of circulating tumor B-cells was not associated with presence of mRNA in plasma.

*BCL2*, *MYC*, *CCND2*, *BCL6* and *LMO2* expression was studied in tumor tissue from 12 DLBCL patients of our series using real-time PCR. *FN1* expression was not analyzed due to that the 12 tissues obtained showed absence of *FN1* mRNA in plasma. mRNA of all genes was detectable in all tissue samples. We analyzed the possible associations between levels of mRNA in tumor tissues and detection in plasma for each marker (Figure S2). BCL2 mRNA in plasma was detected in those patients with higher levels of *BCL2* expression in tumors (*p* = 0.021; Mann-Whitney test). We found no more associations with the others markers studied. Moreover, we observed an association between high levels of *BCL2* expression in tumors and high IPI score, and a trend to signification with patient who died (*p* = 0.007 and *p* = 0.086, respectively: Mann-Whitney test).

### Tumor-Related mRNA in Plasma and Patients' Survival

#### Overall survival

**Figure 1:**
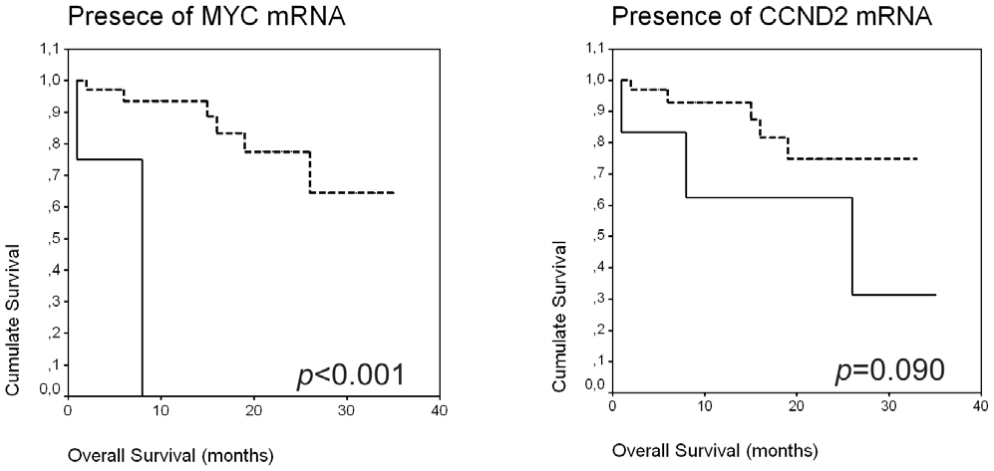
Kaplan-Meier OS curves in relation to presence of MYC and CCND2 mRNA in plasma.

Patients with presence of *MYC* and *CCND2* mRNA in plasma had shorter survival than patients without them (*p<*0.001 and *p* = 0.09, respectively; Kaplan-Meier test; Figure 1). No significant difference in OS was found for the rest of mRNAs analyzed. When we eliminated the censured cases with a follow-up below 12 months, we found a statistical significance for *BCL2* and *MYC* mRNA (*p* = 0.003 for both).

#### Progression-free survival

The Kaplan-Meier test only revealed a trend to significant association between detection of *MYC* mRNA in plasma and patients with short PFS (*p* = 0.099; Kaplan-Meier test). Nor did this change, when we eliminated the censured cases with follow-up below 12 months.

#### Tumor-related mRNA in plasma and overall survival in low-risk patients according to IPI

**Figure 2:**
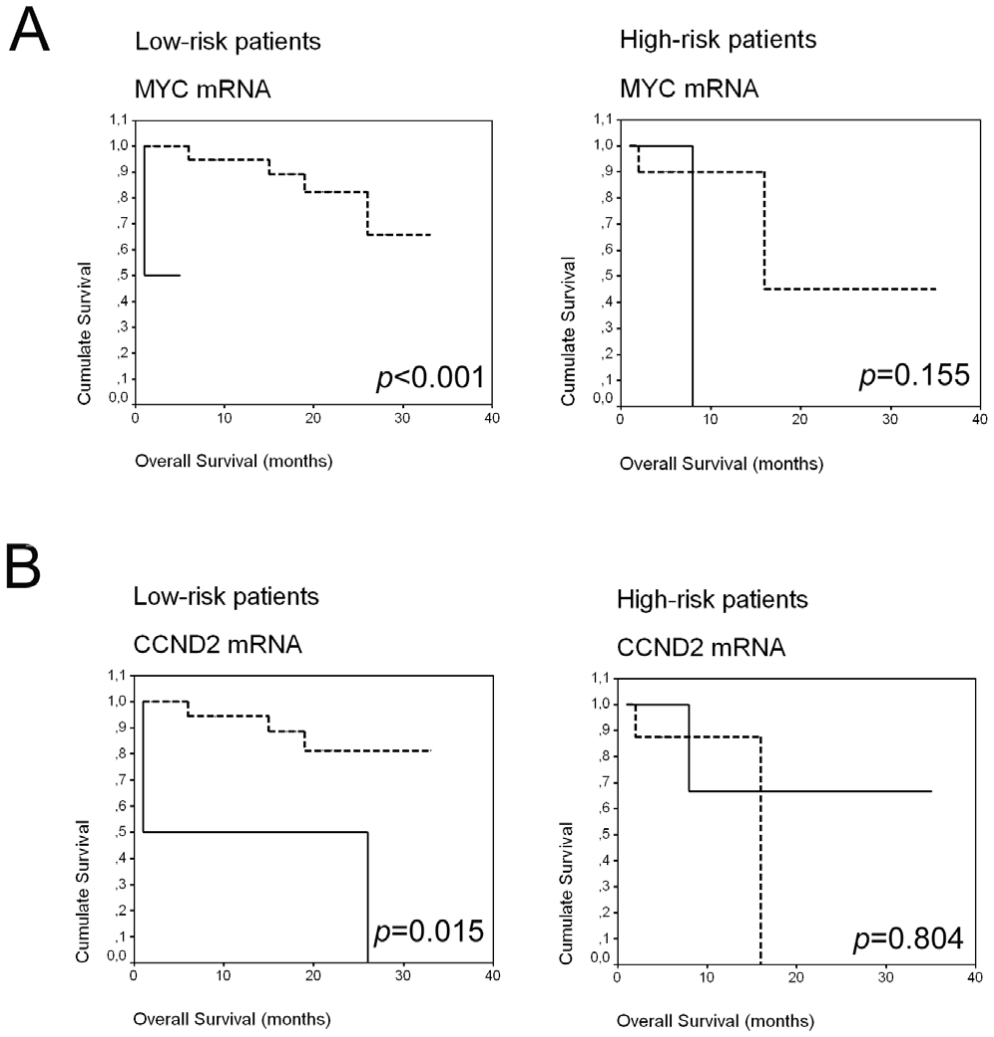
Kaplan-Meier OS curves in relation to presence of MYC (A) and CCND2 (B) mRNA in plasma from patients classified according to IPI.

In our patients with low-risk IPI (0–2 IPI score; N = 28), a significant difference, with worse OS when *CCND2* and *MYC* mRNA were present in plasma, was observed (*p* = 0.015 and *p*<0.001, respectively; Kaplan-Meier test; Figure 2). In these patients, presence of *MYC* mRNA in plasma was associated with partial response to treatment (*p* = 0.011, χ^2^test). We found an association close to significance between high LDH levels and presence of *CCND2*, *MYC* or *BCL6* mRNA in plasma (*p* = 0.068 for the 3 mRNA, χ^2^test). The relationship with high LDH levels was significant with presence of at least one unfavorable molecular marker (*p* = 0.023, χ^2^test). Presence of at least one favorable molecular marker was associated with absence of extranodal involvement (*p* = 0.039, χ^2^test). Finally, all low-risk patients with *CCND2* mRNA in plasma died during the follow-up period (*p* = 0.002, χ^2^test).

### Tumor-Related mRNA in Plasma and Treatment

Our DLBCL patients were treated with rituximab-CHOP and 66% of them had complete response to treatment. Presence of *BCL2* and *MYC* mRNA in plasma showed a trend to significant association with partial response in patients with DLBCL (*p* = 0.07 for both; χ^2^test; Table 1). When we analyzed the presence of at least one marker of poor prognosis, the association with partial response was stronger (*p* = 0.059, χ^2^test). Presence of *LMO2* mRNA in plasma was not significant (*p* = 0.13, χ^2^test), but, interestingly, all positive cases had complete response. The rest of mRNA analyzed showed no significant associations with response to treatment.

**Table 1:** Associations between clinicopathological characteristics and presence of mRNAs in plasma from patients with DLBCL (χ^2^ test).

	*BCL2* mRNA			*CCND2* mRNA			*MYC* mRNA			*LMO2* mRNA		
	+ (%)	− (%)	*p*	+ (%)	− (%)	*p*	+ (%)	− (%)	*p*	+ (%)	− (%)	*p*
**Age**												
<60	2.5	31.7	NS	0	34.2	**0.056**	2.5	31.7	NS	7.3	26.8	**0.070**
≥60	7.3	58.5		14.6	51.2		7.3	58.5		2.5	63.4	
**Grade**												
Intermediate	2.4	39	NS	0	41.5	**0.026**	0	41.5	**0.076**	4.9	36.6	NS
High	7.3	51.3		14.6	43.9		9.7	48.8		4.9	53.6	
**LNA**												
<4	5.9	70.6	NS	8.8	67.7	NS	5.9	70.6	NS	8.8	67.7	NS
≥4	5.9	17.6		8.8	14.7		5.9	17.6		2.9	20.6	
**EI**												
Absence	4.9	43.9	NS	2.4	46.4	NS	4.9	43.9	NS	7.3	41.5	NS
Presence	4.9	46.3		12.2	39		4.9	46.3		2.4	48.8	
**BMI**												
Negative	8.6	82.9	NS	5.7	85.7	NS	5.7	85.7	NS	11.4	80	NS
Positive	2.8	5.7		2.9	5.7		2.9	5.7		0	8.6	
**LDH**												
Normal	4.9	53.6	NS	2.4	56.1	**0.024**	2.4	56.1	NS	9.8	48.8	**0.076**
High	4.9	36.6		12.2	29.3		7.3	34.2		0	41.4	
**IPI**												
Low-risk (0–2)	2.4	65.9	**0.050**	4.9	63.4	**0.046**	4.9	63.4	NS	7.3	61	NS
High-risk (3–5)	7.3	24.4		9.8	21.9		4.9	26.8		2.4	29.3	
**RT**												
Complete	2.5	63.4	**0.070**	7.3	58.5	NS	2.5	63.4	**0.070**	9.8	56.1	NS
Partial	7.3	26.8		7.3	26.9		7.3	26.8		0	34.1	
**LRS**												
Without disease	0	67.7	**0.008**	6.5	61.3	NS	0	67.7	**0.034**	9.7	58.1	NS
With disease	9.7	22.6		3.2	29		6.5	25.8		0	32.2	
**VSG**												
Normal	0	43.3	NS	0	43.3	**0.060**	0	43.3	NS	3.3	40	NS
Anormal	6.7	50		13.3	43.3		10	46.7	NS	6.7	50	
**Albumine**												
Nomal	5.1	82.1	**0.019**	10.3	76.9	NS	5.1	82.1	**0.019**	7.7	79.5	NS
<4	5.1	7.7		5.1	7.7		5.1	7.7		2.6	10.2	

+: presence in plasma; −: absence in plasma; LNA: number of lymph nodes affected; EI: extranodal involvement; BMI: bone marrow infiltrated; RT: response to treatment; LRS: last revision status.

In plasma from patients with complete response to treatment, we observed that patients with presence of *CCND2*, *BCL2* or *MYC* mRNA had shorter PFS than patients without these markers (*p* = 0.037, *p* = 0.016, *p* = 0.016, respectively; Kaplan-Meier test; Figure S3).

### Correlation of Tumor-Related mRNA in Plasma with Clinico-Pathological Characteristics

We analyzed the possible associations between our molecular markers and the clinico-pathological parameters of the tumors (Table 1). Presence of *BCL2*, *CCND2* and *MYC* mRNA in plasma was associated with several characteristics of poor prognosis. When presence of LMO2 mRNA was analyzed, we only observed a trend to significant associations with characteristics of good prognosis. *BCL6* and *FN1* mRNA were not associated with any parameter.

### Immunohistochemistry Analysis

**Figure 3:**
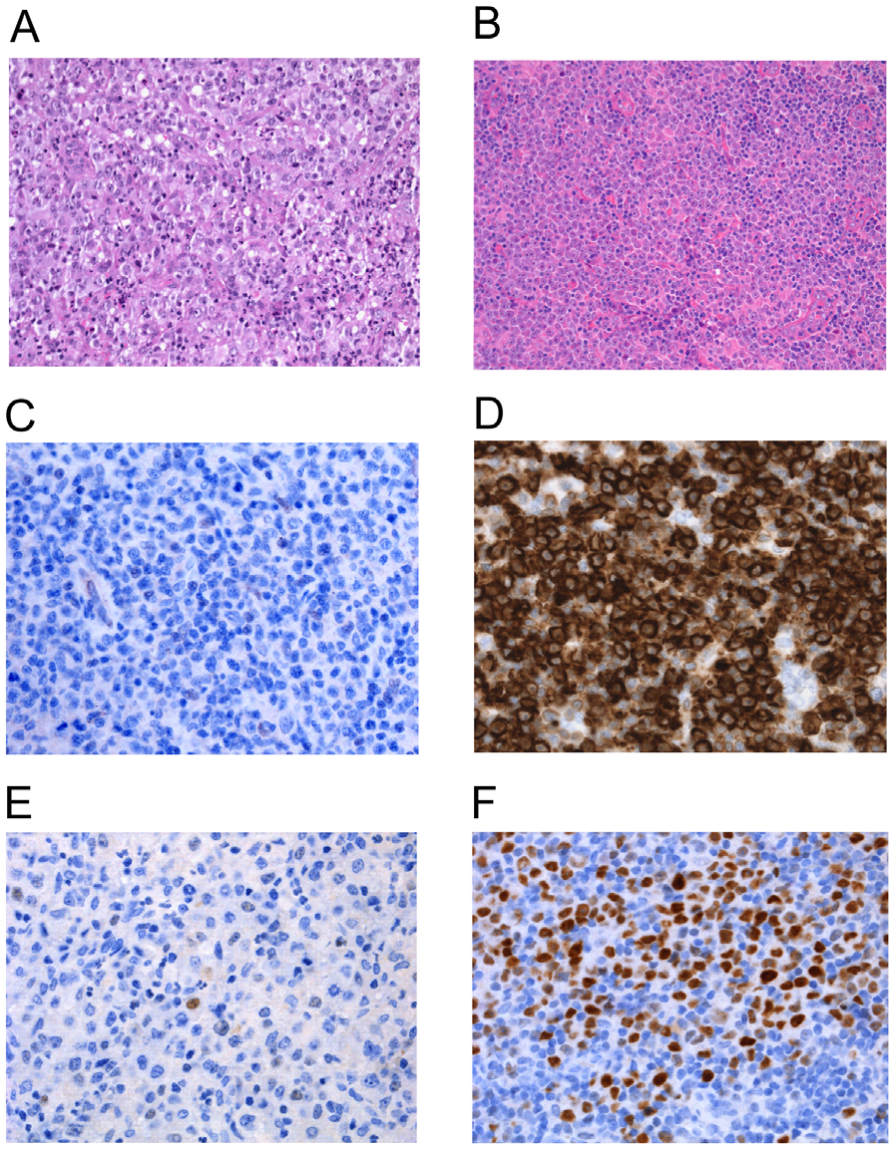
Morphologic features and IHC expression in representative DLBCL patients. A) DLBCL anaplastic variant (H&E staining x20). B) Lymph node section (H&E staining x20). IHC for BCL2 (x40) showing: C) Neoplastic cells negative for BCL2 and D) Strong cytoplasmic staining. IHC for BCL6 (x40) showing: E) BCL6 negative case and F) BCL6 positive case with strong nuclear staining.

Immunohistochemistry analysis for BCL2 and BCL6 was performed on tumor tissue from 24 and 26 DLBCL patients, respectively, in our series (Figure 3). BCL2 and BCL6 expression were detected in 54% and 89% of the tumors, respectively. BCL2 and BCL6 expression were not associated with detection of *BCL2* and *BCL6* mRNA in plasma, respectively. However, absence of BCL6 expression in tumors was associated with presence of *BCL2*, *CCND2* and *MYC* mRNA in plasma (*p* = 0.009, *p* = 0.001 and *p* = 0.009, respectively; χ^2^test). Moreover, absence of BCL6 expression in tumors was associated with extranodal involvement (*p* = 0.047), high IPI score (*p* = 0.006) and “B” symptoms (*p* = 0.002), and with short overall survival (*p* = 0.011; χ^2^ and Kaplan-Meier test).

### Presence of Tumor-Related mRNA in Plasma from Patients after Chemotherapy Treatment

Plasma obtained from 13 patients with DLBCL after treatment was analyzed. Presence of *BCL2*, *CCND2*, *MYC* and *BCL6* mRNA post-treatment was detected in one patient, who had partial response to treatment and subsequently died (*p* = 0.015 for the four mRNA; χ^2^test). *LMO2* mRNA in post-treatment plasma was detected in two patients who died (*p*<0.001, χ^2^test). In the patients with complete response, presence of *BCL2*, *CCND2*, *MYC* and *BCL6* mRNA detected before treatment was lost in post-treatment samples.

## Discussion

*LMO2*, *BCL6* and *FN1* mRNA in lymphoma correlated with prolonged survival [2], but when we detected them in plasma, no differences with healthy controls or associations with favorable outcome were found, except for *LMO2* mRNA. This could be explained because the release of free nucleic acids into plasma for tumor cells is a characteristic of poor outcome, which might overcome the good prognostic value of these mRNA within the lymphoma cells. When we studied mRNA in plasma of poor-prognosis genes in tumors (*MYC*, *CCND2* and *BCL2*) [2], patients with presence of these mRNA in plasma have clinical and biochemical features indicative of poor prognosis and short survival. These results suggest that unfavorable molecular markers described in tumors [2], mainly *MYC* mRNA, could similarly be markers of poor outcome if they are found in plasma. Our results suggested that the markers found in plasma from DLBCL relating to poor outcome had lower prognostic value in B-cell lymphoma with slow proliferation (FL) and, overall, with HL.

One hypothesis about the origin of plasma mRNA is that it is released by circulating tumor cells [14], [15]. However, we only found one patient with presence of circulating tumor B-cell monoclonality. Recently, several studies have reported that mRNA is released by an active mechanism in tumor cells [9]–[11].

Patients with presence of *BCL2*, *CCND2*, *BCL6* and *LMO2* mRNA in plasma showed higher expression levels for each gene in tumor tissue, although we only observed a significant association for BCL2 mRNA. In the literature, approximately 47% to 58% of DLBCL tumors express BCL2 protein and 56% to 70% express BCL6 protein [16]. Our results are similar, although BCL6 is more expressed in our series. We found no association between levels of BCL2 and BCL6 protein in tumors and presence of *BCL2* and *BCL6* mRNA in plasma. In a recent study, high expression of BCL6 in tumors analyzed immunohistochemically was associated with better overall survival in patients treated with CHOP and with R-CHOP [17]. In our study, BCL6 over-expression in tumors was a marker of favorable outcome. Curiously, expression of BCL6 protein was related to absence of the unfavorable markers we studied in plasma. Thus, presence of unfavorable mRNAs in plasma is found in patients with poor outcome.

The IPI is established as one of the best predictors of outcome [18]. However, variable survival of patients with identical prognostic scores is found. In our study, presence of unfavorable mRNA in plasma from patients with low risk (0–2 IPI score) was associated with poor-prognosis characteristics and shorter overall survival. Our results suggest that plasma mRNA could be used as a molecular marker that identifies patients with poor prognosis in a subset of patients clinically classified in a favorable outcome. Tumors with similar cell phenotypes, classified with the same IPI score, could have different capacities for active liberation of mRNA into plasma, which could be interpreted as a more aggressive characteristic of some of these lymphomas. Thus, finding molecular features such as extracellular mRNAs of unfavorable genes, which identify patients with poor outcome within a low IPI, is an important objective.

An improvement in survival of patients with DLBCL when rituximab was added to CHOP chemotherapy has led to its use as the new standard of care [1]. When several prognostic markers were re-evaluated in the rituximab era, the prognostic values appeared to be mitigated or transformed, as happened with BCL6 behavior [2]. For this reason, identification of new prognostic factors in patients receiving rituximab is important. Our results suggest that detection of unfavorable markers in plasma, such as *MYC* or *BCL2* mRNA, could be possible markers of poor outcome and no response to treatment in the rituximab era. In addition, disappearance of circulating mRNAs after chemotherapy was observed in patients with complete response, while their persistence was associated with partial response and death. These results could indicate an active tumor origin of mRNAs in plasma and could introduce the molecular remission concept, just as has been established in the follow-up of some leukemias.

In conclusion, the presence of circulating nucleic acids in patients with DLBCL could lead to the development of non-invasive methods, useful for distinguishing tumors with different clinical features, treatment response and outcome in the rituximab era. Only the markers with unfavorable prognosis in tumors kept their prognostic value in plasma. In particular, *MYC* mRNA could be a marker of poor outcome and partial response to R-CHOP. Moreover, our unfavorable markers in plasma showed differences between lymphoma subtypes: they were more characteristic of B-cell tumors with high proliferation. Finally, in patients clinically classified in the good prognosis group by IPI, presence of mRNA in plasma, such as *MYC* and *CCND2* mRNA, could indicate a subgroup of patients with poor outcome.

## Supporting Information

Figure S1 Genescan analysis of DNA isolated from MNC amplified with FR3 primers of IgH. A: monoclonal sample with a single peak. B: polyclonal sample with many IgH PCR products. C: positive monoclonal control. D: polyclonal control. E: negative sample without DNA.
(0.20 MB TIF)

Figure S2 Box plots showing the relationship between levels of mRNA in tumor tissues (Normalized Relative Ratio) and detection in plasma for each marker studied. The graphs show the quartiles 25, 50, and 75, values lower than 1.5 box lengths, and the outliers.
(0.05 MB TIF)

Figure S3 Kaplan-Meier PFS curves in relation to presence of BCL2, CCND2 and MYC mRNA in plasma of DLBCL patients with complete response to chemotherapy.
(0.05 MB TIF)

Table S1 (0.03 MB DOC)

Table S2 (0.20 MB DOC)

Table S3 (0.03 MB DOC)

## References

[1] Coiffier B, Lepage E, Briere J, Herbrecht R, Tilly H (2002). CHOP chemotherapy plus rituximab compared with CHOP alone in elderly patients with diffuse large-B-cell lymphoma.. N Engl J Med.

[2] Lossos IS, Morgensztern D (2006). Prognostic biomarkers in diffuse large B-cell lymphoma.. J Clin Oncol.

[3] Lossos IS, Czerwinski DK, Alizadeh AA, Wechser MA, Tibshirani R (2004). Prediction of survival in diffuse large-B-cell lymphoma based on the expression of six genes.. N Engl J Med.

[4] Garcia JM, Garcia V, Silva J, Peña C, Dominguez G (2006). Extracellular tumor DNA in plasma and overall survival in breast cancer patients.. Genes Chromosomes Cancer.

[5] Garcia V, Garcia JM, Pena C, Silva J, Domínguez G (2008). Free circulating mRNA in plasma from breast cancer patients and clinical outcome.. Cancer Lett.

[6] Gandhi MK, Lambley E, Burrows J, Dua U, Elliott S (2006). Plasma Epstein-Barr virus (EBV) DNA is a biomarker for EBV-positive Hodgkin's lymphoma.. Clin Cancer Res.

[7] Frickhofen N, Muller E, Sandherr M, Binder T, Bangerter M (1997). Rearranged Ig heavy chain DNA is detectable in cell-free blood samples of patients with B-cell neoplasia.. Blood.

[8] Dasi F, Lledo S, Garcia-Granero E, Ripoll R, Marugán M (2001). Real-time quantification in plasma of human telomerase reverse transcriptase (hTERT) mRNA: a simple blood test to monitor disease in cancer patients.. Lab Invest.

[9] Garcia JM, Garcia V, Pena C, Domínguez G, Silva J (2008). Extracellular plasma RNA from colon cancer patients is confined in a vesicle-like structure and is mRNA-enriched.. RNA.

[10] Valadi H, Ekstrom K, Bossios A, Sjöstrand M, Lee JJ (2007). Exosome-mediated transfer of mRNAs and microRNAs is a novel mechanism of genetic exchange between cells.. Nat Cell Biol.

[11] Skog J, Würdinger T, van Rijn S, Meijer DH, Gainche L (2008). Glioblastoma microvesicles transport RNA and proteins that promote tumour growth and provide diagnostic biomarkers.. Nat Cell Biol.

[12] Van Dongen JJ, Langerak AW, Brüggemann M, Evans PA, Hummel M (2003). Design and standardization of PCR primers and protocols for detection of clonal immunoglobulin and T-cell receptor gene recombinations in suspect lymphoproliferations: report of the BIOMED-2 Concerted Action BMH4-CT98-3936.. Leukemia.

[13] Tibiletti MG, Martin V, Bernasconi B, Del Curto B, Pecciarini L (2009). BCL2, BCL6, MYC, MALT1, and BCL10 rearrangements in nodal diffuse large B-cell lymphomas: a multicenter evaluation of a new set of fluorescent in situ hybridization probes and correlation with clinical outcome.. Hum Pathol.

[14] Shapiro B, Chakrabarty M, Cohn EM, Leon SA (1983). Determination of circulating DNA levels in patients with benign or malignant gastrointestinal disease.. Cancer.

[15] Silva JM, Rodriguez R, Garcia JM, Munoz C, Silva J (2002). Detection of epithelial tumour RNA in the plasma of colon cancer patients is associated with advanced stages and circulating tumour cells.. Gut.

[16] Lossos IS, Morgensztern D (2006). Prognostic biomarkers in diffuse large B-cell lymphoma.. J Clin Oncol.

[17] Seki R, Ohshima K, Fujisaki T, Uike N, Kawano F Cancer Sci 2009.

[18] A predictive model for aggressive Non-Hodgkin's lymphoma. The international Non-Hodgkin's lymphoma prognostic factors project.. N Engl J Med 1993;.

