# Supplementary figures and images for: Extracellular Tumor-Related mRNA in Plasma of Lymphoma Patients and Survival Implications
Source: PLoS One. 2009 Dec 15;4(12):e8173. doi: 10.1371/journal.pone.0008173 (PMC2788245; doi:10.1371/journal.pone.0008173)

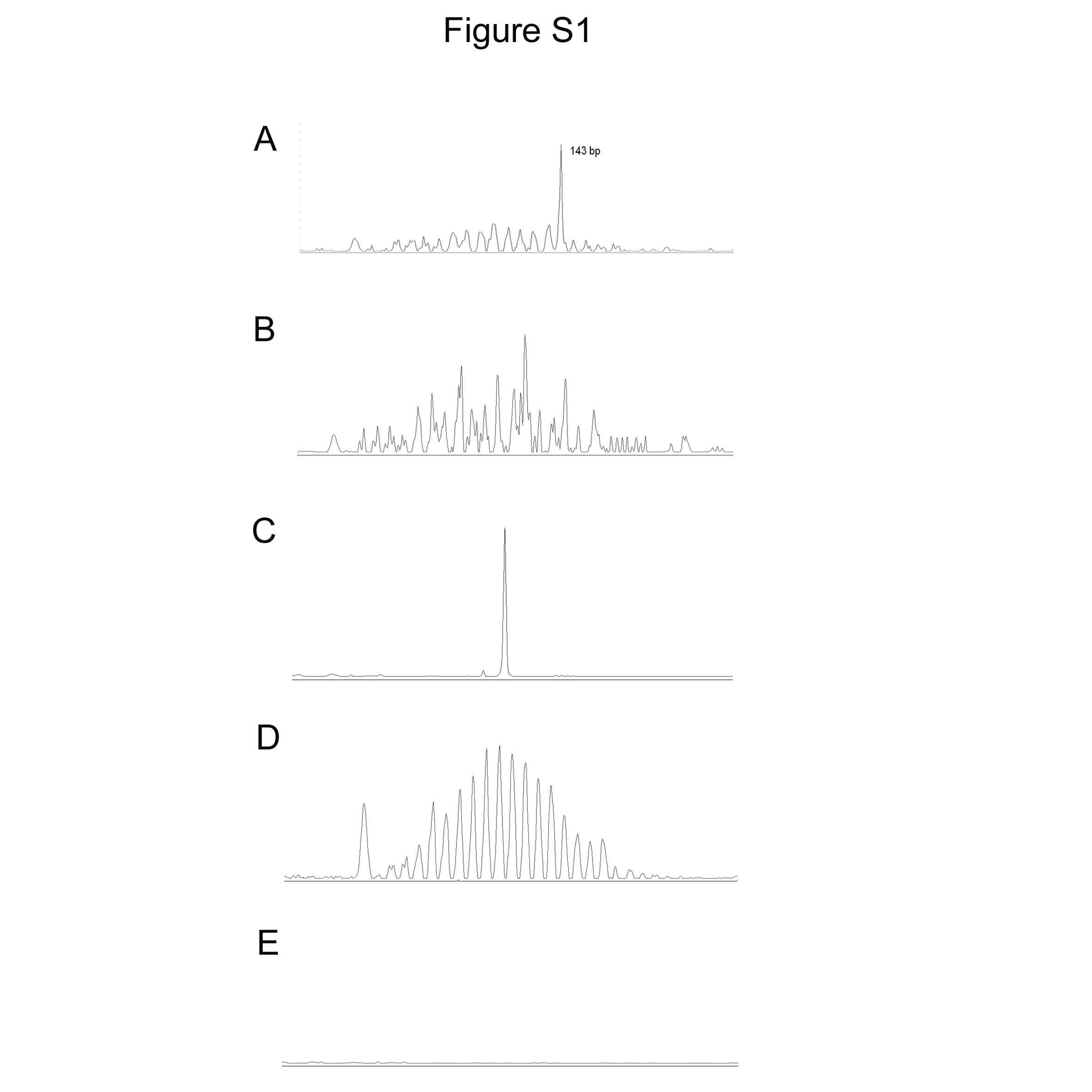

Supplement: Figure S1 — Genescan analysis of DNA isolated from MNC amplified with FR3 primers of IgH. A: monoclonal sample with a single peak. B: polyclonal sample with many IgH PCR products. C: positive monoclonal control. D: polyclonal control. E: negative sample without DNA. (0.20 MB TIF) [file pone.0008173.s001.tif]

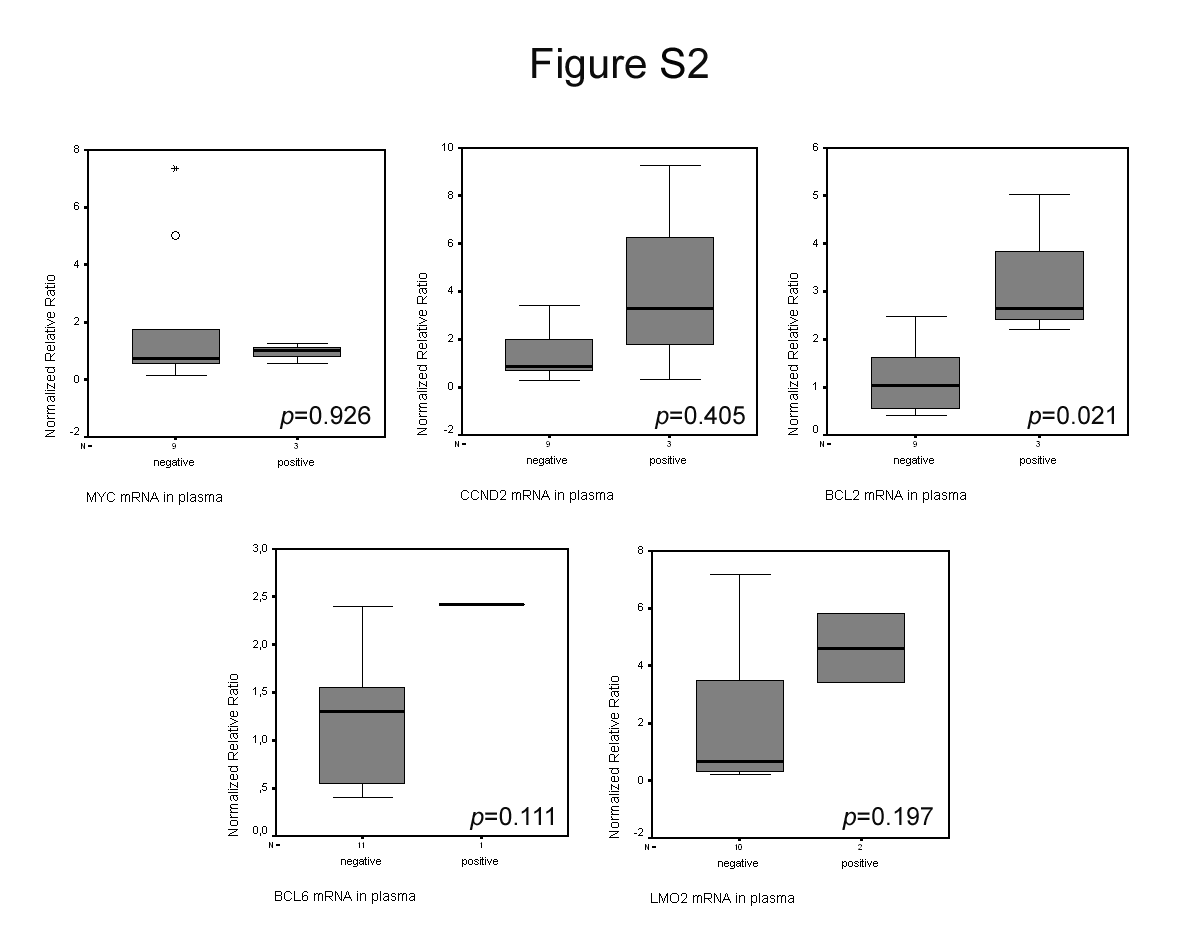

Supplement: Figure S2 — Box plots showing the relationship between levels of mRNA in tumor tissues (Normalized Relative Ratio) and detection in plasma for each marker studied. The graphs show the quartiles 25, 50, and 75, values lower than 1.5 box lengths, and the outliers. (0.05 MB TIF) [file pone.0008173.s002.tif]

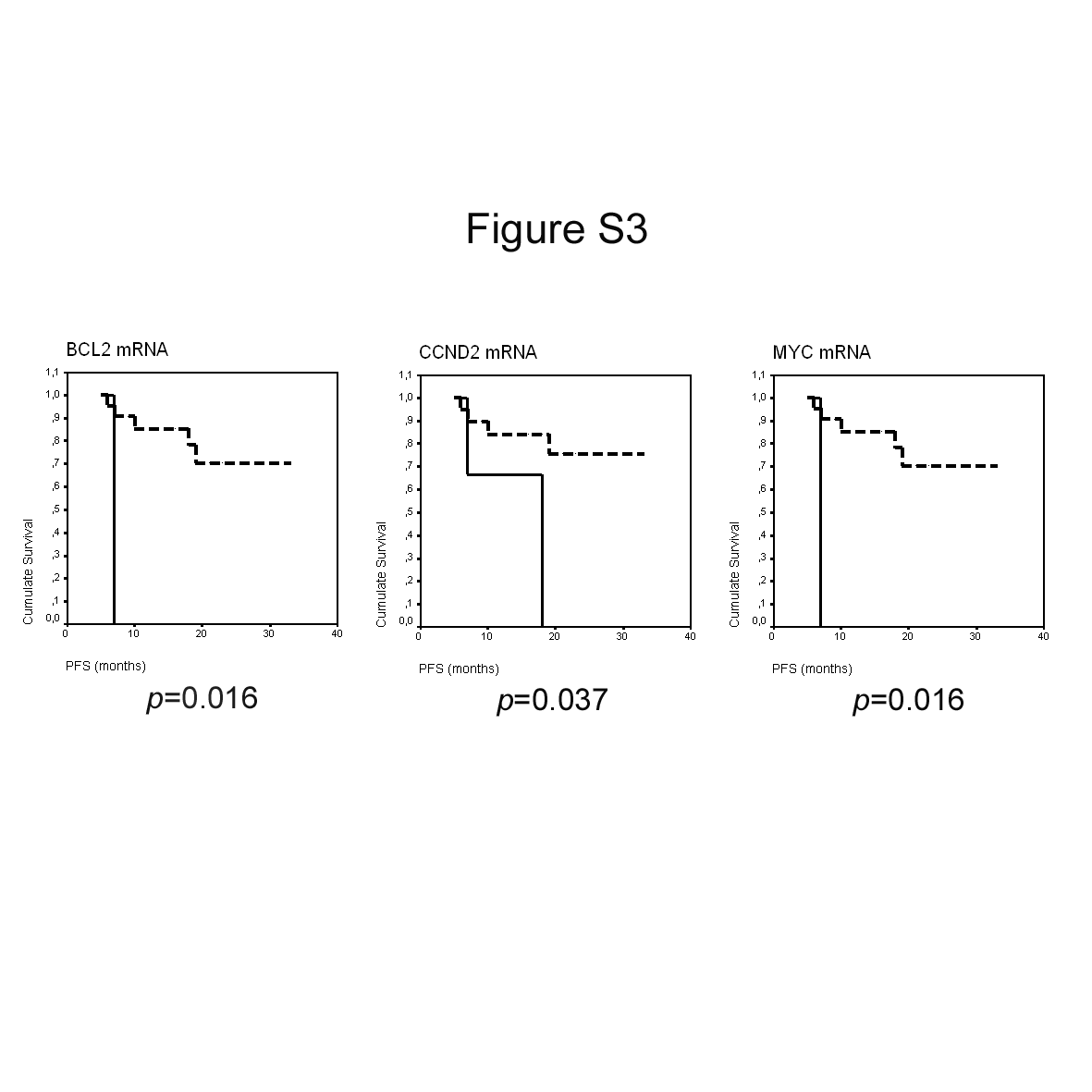

Supplement: Figure S3 — Kaplan-Meier PFS curves in relation to presence of BCL2, CCND2 and MYC mRNA in plasma of DLBCL patients with complete response to chemotherapy. (0.05 MB TIF) [file pone.0008173.s003.tif]
