# Supplementary material for: Extracellular Tumor-Related mRNA in Plasma of Lymphoma Patients and Survival Implications
Source: PLoS One. 2009 Dec 15;4(12):e8173. doi: 10.1371/journal.pone.0008173 (PMC2788245; doi:10.1371/journal.pone.0008173)
Supplement: Table S1 — (0.03 MB DOC) [file pone.0008173.s004.doc]

**Table S1**. Sequences and annealing temperatures (AT) for each primer used.

| **mRNA** | **Primers Sequence** | **AT (ºC)** |
| --- | --- | --- |
| *CCND2* | 5´GTGTGATGCCATATCAAGTCC3´F  5´TCGCATACACTGATCATGC3´R | 55 |
| *BCL2* | 5´GAGTAAATCCATGCACCTAAACC3´F  5´TGCAAATTCTACCTTGGAGGG3´R | 61 |
| *MYC* | 5´TGGATTTTTTTCGGGTAGTGG3´F  5´GTCGTAGTCGAGGTCATAGTTCC3´R | 59 |
| *BCL6* | 5´CCTGTGAAATCTGTGGCACCCG3´F  5´CGCAGCTGGCTTTTGTGACGG3´R | 61 |
| *LMO2* | 5´CCTCTACTACAAACTGGGCCG3´F  5´TCTCATAGGCACGAATCCG3´R | 60 |
| *FN1* | 5´GAGTGTGTGTGTCTTGGTAATGG3´F  5´CCACGTTTCTCCGACCAC3´R | 59 |
| *PGK1* | 5´AGCTGCTGGGTCTGTCATCC3´F  5´AGCTTCTATTTTGGCTGGCTCG3´R | 60 |
