# Supplementary material for: Extracellular Tumor-Related mRNA in Plasma of Lymphoma Patients and Survival Implications
Source: PLoS One. 2009 Dec 15;4(12):e8173. doi: 10.1371/journal.pone.0008173 (PMC2788245; doi:10.1371/journal.pone.0008173)
Supplement: Table S2 — (0.20 MB DOC) [file pone.0008173.s005.doc]

**Table S2. Ct values of DLBCL patients and healthy controls (HC) samples for each plasma mRNA studied. NS, negative sample.**

| **SAMPLES** | **BCL2 mRNA** | **CCND2 mRNA** | **MYC mRNA** | **BCL6 mRNA** | **LMO2 mRNA** | **FN1 mRNA** |
| --- | --- | --- | --- | --- | --- | --- |
| DLBCL patient 1 | NS | NS | NS | NS | NS | NS |
| DLBCL patient 2 | NS | NS | NS | NS | NS | NS |
| DLBCL patient 3 | NS | NS | NS | NS | NS | NS |
| DLBCL patient 4 | NS | NS | NS | NS | NS | NS |
| DLBCL patient 5 | NS | NS | NS | NS | NS | NS |
| DLBCL patient 6 | NS | NS | NS | NS | NS | NS |
| DLBCL patient 7 | NS | NS | NS | NS | 32,64 | NS |
| DLBCL patient 8 | NS | NS | NS | NS | NS | NS |
| DLBCL patient 9 | NS | NS | NS | NS | NS | NS |
| DLBCL patient 10 | NS | NS | NS | NS | NS | NS |
| DLBCL patient 11 | 29,24 | 29,66 | 31,38 | NS | 30,41 | NS |
| DLBCL patient 12 | NS | NS | NS | NS | NS | NS |
| DLBCL patient 13 | NS | 32,71 | NS | NS | NS | NS |
| DLBCL patient 14 | NS | NS | NS | NS | NS | NS |
| DLBCL patient 15 | NS | NS | NS | NS | NS | NS |
| DLBCL patient 16 | NS | NS | NS | 30,38 | NS | NS |
| DLBCL patient 17 | NS | NS | NS | NS | NS | NS |
| DLBCL patient 18 | NS | NS | NS | NS | NS | NS |
| DLBCL patient 19 | NS | NS | NS | NS | NS | NS |
| DLBCL patient 20 | NS | NS | NS | NS | NS | NS |
| DLBCL patient 21 | NS | NS | NS | NS | NS | NS |
| DLBCL patient 22 | NS | NS | NS | NS | NS | NS |
| DLBCL patient 23 | NS | NS | NS | NS | 33,03 | NS |
| DLBCL patient 24 | NS | NS | NS | NS | NS | NS |
| DLBCL patient 25 | NS | NS | NS | NS | NS | NS |
| DLBCL patient 26 | NS | NS | NS | NS | NS | NS |
| DLBCL patient 27 | NS | 30,75 | 33,21 | NS | NS | NS |
| DLBCL patient 28 | NS | NS | NS | NS | NS | NS |
| DLBCL patient 29 | NS | 32,38 | NS | NS | NS | NS |
| DLBCL patient 30 | NS | NS | NS | NS | NS | NS |
| DLBCL patient 31 | NS | NS | NS | NS | NS | NS |
| DLBCL patient 32 | 31,24 | NS | NS | NS | NS | NS |
| DLBCL patient 33 | 29,86 | NS | 33,43 | 31,53 | NS | NS |
| DLBCL patient 34 | NS | NS | NS | NS | 33,6 | NS |
| DLBCL patient 35 | NS | NS | NS | NS | NS | NS |
| DLBCL patient 36 | NS | NS | NS | NS | NS | NS |
| DLBCL patient 37 | 31,74 | 29,26 | 32,57 | NS | NS | NS |
| DLBCL patient 38 | NS | NS | NS | NS | NS | 33,2 |
| DLBCL patient 39 | NS | NS | NS | NS | NS | NS |
| DLBCL patient 40 | NS | 33,99 | NS | NS | NS | NS |
| DLBCL patient 41 | NS | NS | NS | NS | NS | NS |
| DLBCL patient 42 | NS | NS | NS | NS | NS | NS |
| HC 1 | NS | NS | NS | NS | NS | NS |
| HC 2 | NS | NS | NS | NS | NS | NS |
| HC 3 | NS | NS | NS | NS | NS | NS |
| HC 4 | NS | NS | NS | NS | NS | NS |
| HC 5 | NS | NS | NS | NS | NS | NS |
| HC 6 | NS | NS | NS | NS | NS | NS |
| HC 7 | NS | NS | NS | NS | NS | NS |
| HC 8 | NS | NS | NS | NS | NS | NS |
| HC 9 | NS | NS | NS | NS | NS | NS |
| HC 10 | NS | NS | NS | NS | NS | NS |
| HC 11 | NS | NS | NS | NS | NS | NS |
| HC 12 | NS | NS | NS | NS | NS | NS |
| HC 13 | NS | NS | NS | NS | NS | NS |
| HC 14 | NS | NS | NS | NS | NS | NS |
| HC 15 | NS | NS | NS | NS | NS | NS |
| HC 16 | NS | NS | NS | NS | NS | NS |
| HC 17 | NS | NS | NS | NS | NS | NS |
| HC 18 | NS | NS | NS | NS | NS | NS |
| HC 19 | NS | NS | NS | NS | NS | NS |
| HC 20 | NS | NS | NS | NS | NS | NS |
| HC 21 | NS | NS | NS | NS | NS | NS |
| HC 22 | NS | NS | NS | NS | NS | NS |
| HC 23 | NS | NS | NS | NS | NS | NS |
| HC 24 | NS | NS | NS | NS | NS | NS |
| HC 25 | NS | NS | NS | NS | NS | NS |
| HC 26 | NS | NS | NS | NS | NS | NS |
| HC 27 | NS | NS | NS | NS | NS | NS |
| HC 28 | NS | NS | NS | NS | NS | NS |
| HC 29 | NS | NS | NS | NS | NS | NS |
| HC 30 | NS | NS | NS | NS | NS | NS |
| HC 31 | NS | NS | NS | NS | NS | NS |
| HC 32 | NS | NS | NS | NS | NS | NS |
| HC 33 | NS | NS | NS | NS | NS | NS |
| HC 34 | NS | NS | NS | NS | NS | NS |
| HC 35 | NS | NS | NS | NS | NS | NS |
| HC 36 | NS | NS | NS | NS | NS | NS |
| HC 37 | NS | NS | NS | NS | NS | NS |
| HC 38 | NS | NS | NS | NS | NS | NS |
| HC 39 | NS | NS | NS | NS | NS | NS |
| HC 40 | NS | NS | NS | NS | NS | NS |
| HC 41 | NS | NS | NS | NS | NS | NS |
| HC 42 | NS | NS | NS | NS | NS | NS |
| HC 43 | NS | NS | NS | NS | NS | NS |
| HC 44 | NS | NS | NS | NS | NS | NS |
| HC 45 | NS | NS | NS | NS | NS | NS |
| HC 46 | NS | NS | NS | NS | NS | NS |
| HC 47 | NS | NS | NS | NS | NS | NS |
| HC 48 | NS | 33,35 | NS | NS | NS | NS |
| HC 49 | NS | NS | NS | NS | NS | NS |
| HC 50 | NS | NS | NS | NS | NS | NS |
