# Supplementary material for: Extracellular Tumor-Related mRNA in Plasma of Lymphoma Patients and Survival Implications
Source: PLoS One. 2009 Dec 15;4(12):e8173. doi: 10.1371/journal.pone.0008173 (PMC2788245; doi:10.1371/journal.pone.0008173)
Supplement: Table S3 — (0.03 MB DOC) [file pone.0008173.s006.doc]

**Table S3.** Detection of mRNA (%) in plasma from the different series of patients studied.

|  | ***CCND2*** | ***BCL2*** | ***CMYC*** | ***BCL6*** | ***LMO2*** | ***FN1*** |
| --- | --- | --- | --- | --- | --- | --- |
| **DLBCL** | 14.3 | 9.5 | 9.5 | 4.8 | 9.5 | 2.4 |
| **FL** | 8 | 4 | 0 | 4 | 4 | 0 |
| **HDK** | 6.2 | 0 | 0 | 0 | 6.2 | 6.2 |
| **Controls** | 2 | 0 | 0 | 0 | 0 | 0 |
